# Repeated cultivation: non-cell disruption extraction of astaxanthin for *Haematococcus pluvialis*

**DOI:** 10.1038/srep20578

**Published:** 2016-02-03

**Authors:** Han Sun, Bin Guan, Qing Kong, Zhaoyan Geng, Ni Wang

**Affiliations:** 1 School of Food Science and Engineering, Ocean University of China, Qingdao, Shandong 266003, China

## Abstract

The operation of cell disruption is indispensable but cost much in microalgae industry. To be simplified, two different reaction mechanisms await in the cell to respond to moderated or stressed environment. The physical and chemical changes of enzyme and turgor pressure of cell in this conversion play an important role in the enhancement of biomass and metabolites. Repeated turgor pressure (based on the structure and mechanics of cell wall) and converted enzyme system (based on photosynthesis) were used to loosen cell wall and then repeated cultivation of *Haematococcus pluvialis* for astaxanthin extraction was proposed. There was no significant difference of extraction yield between the broken cell (94.75 ± 3.13%) and non-broken cell (92.32 ± 3.24%) treated by the repeated cultivation. Meanwhile, fed-batch culture according to the relationship among pH and nutrient concentration was used to enhance the biomass of *Haematococcus pluvialis* with the dry cell weight of 1.63 ± 0.07 g/L.

Advances in microalgae researches of bioresource and commercial production have been united in the past few decades. Many reports have proved the feasibility and economical efficiency of the bioenergy and metabolites from microalgae^1^,^2^. These have led many institutions and companies to participate in this animate field. However, microalgae industry also has their limitations. The major limitation is high energy demand is badly needed in industrial process and all the spearheads fell on cell wall for the extraction of production recovery, degradation of cell wall and conservation of production quality^3^. Cell disruption, because of the high energy demand and effect on the efficiency of subsequent steps, is the critical step in the traditional process of microalgae industry. Numerous methods are available for cell disruption. Destruction of cell wall is usually achieved by mechanical (e.g., bead milling, high pressure homogenization, high speed homogenization, and ultrasonication) and non-mechanical methods (e.g., enzymatic cell lysis, and chemical cell disruption)^4^,^5^,^6^,^7^,^8^,^9^. The violent physical and chemical reactions could result in nutrient losses like oxidation of lipid through the unsuitable extraction method. The fact that oil droplet in cell hardly contacts with solution and is easy to be oxidized, costs much in industry, thus the unified operations of cell disruption haven’t been achieved in most factories. New ideas have advanced that the relationship between energy consumption and efficiency of cell disruption is the key point to evaluate or even create a method of cell disruption^3^. However, no one is willing to help the industrial process “escape” from cell disruption.

The living microalgae cell typically undergoes a site of tremendous biochemical activities: conversion of light and nutrients to energy, buildup of new tissue, replacement of old tissue, disposal of waste materials and reproduction. For the cell cultivation, energy resource (light) and enzyme reaction display essential roles in biomass and metabolites production. The light intensity and light wavelength dominate the rate of photosynthesis in the aspect of light saturation and the maximum quantum yield^10^. Enzymes are responsible for almost all of the chemical reactions in the living organisms, which are regarded as the potential conversion of energy from light and nutrients. Thus, the proper application of these two factors makes possible that microalgae cell changes the metabolic pathways for high yield of biomass, the production itself and even the cell structure, such as cell wall.

Among microalgae, *Haematococcus pluvialis* is well known for its high bioactive secondary metabolite (astaxanthin) and its complex cell life^11^. *H. pluvialis* undergoes significant morphologic changes from green active cell to red immobile cell, which make the cultivation conditions different to maximize the energy availability according to light efficiency and enzyme reaction^12^,^13^. Astaxanthin is a carotenoid of high biological activity given its potential in food and pharmaceutical industries, as well as in feed industry for the ability to pigment and improve survival rate of aquatic animals. *H. pluvialis* has been used preferably to produce astaxanthin for its high content of astaxanthin at about 5%^11^, which has been reported as the maximal. Furthermore, the structure of astaxanthin from *H. pluvialis* is similar to those in salmon and other aquatic organisms, making astaxanthin more easily to be absorbed. As a result, many efforts have been focused on astaxanthin-rich *H. pluvialis.*

Summarized from the reported researches, all the techniques and methods are based on one-stage or two-stage production process^14^,^15^,^16^. Adjustment of cultivation conditions to different growth stages or different strains of *H. pluvialis* aims at promoting energy absorption and improving enzyme activity to obtain high biomass and production of astaxanthin^17^,^18^,^19^. As shown in Fig. 1, the cultivation of *H. pluvialis* can be simply divided into two stages, the cell growth and astaxanthin accumulation. The green growth stage usually lasts for 9 to 20 days according to the relationship between cell biomass and cell activity. In the astaxanthin accumulation stage, cellular morphology changes to aplanospore with the decreasing nutrients and increasing stress environment, which are different from optimal conditions for vegetative cell growth. Generally, two-stage production process is regarded as the common cultivation process of *H. pluvialis* according to the morphologic changes in the life cycle of *H. pluvialis* which is from green active cell to red immobile cell. In our previous research, mixed-light of blue and white (3:1) expedited the proceeding of green active cell to red immobile cell from 9 days to 6 days^14^. One-stage production process is developed to simplify the operations in two-stage production process with the optimal cultivation conditions, which combines green growth stage and astaxanthin accumulation stage. Theoretically, two-stage performs better than one-stage and that have also been proved by the reported researches^20^. The simplified mind (one stage production) is usually present in the industry for the easy operations in large scale and relative short time. However, complicated mind (two stage production) does not mean the hard operations in industry.

Herein, according to the property of *H. pluvialis* presented in the green growth and astaxanthin accumulation stage, we proposed repeated cultivation (three-stage production process) that will renovate astaxanthin production by *H. pluvialis* in industry (Fig. 1). After 6 days of astaxanthin accumulation, the second growth started when the environment transformed to growth environment for 2 days to induce the germination of *H. pluvialis.* In this period, cell wall was relaxed. Then the cultivation environment returned to the accumulation stage for 1 day. The circle continued until the non-cell disruption was achieved. The meaningfulness of this revised process is that the operation of cell disruption in industry is removed with the same extraction yield as two-stage process.

This study aimed at exploring an impactful repeated cultivation process (three-stage process) to renovate *H. pluvialis* for wiping off cell disruption. The mixed -light of red and white was used to increase the cell biomass and also for pullback of green *H. pluvialis* from red cell to regulate cell wall. Fed-batch culture was applied to obtain high biomass and astaxanthin production according to the changing pH in the growth process. Extraction of astaxanthin of three-stage process was studied compared with broken cell of two-stage process.

## Result

### Effect of lights on cell activity and cell biomass in batch culture

Conversion of light and nutrient to energy is an important part of metabolism of a living cell in chlorophyta. The luminous energy is translated into chemical energy in photochemical reaction center or by light-harvesting complex^21^. The supplement of light and nutrient are important for other processes in metabolism such as buildup of new tissue and disposal of waste materials, as well as for cell biomass.

**Figure 2:**
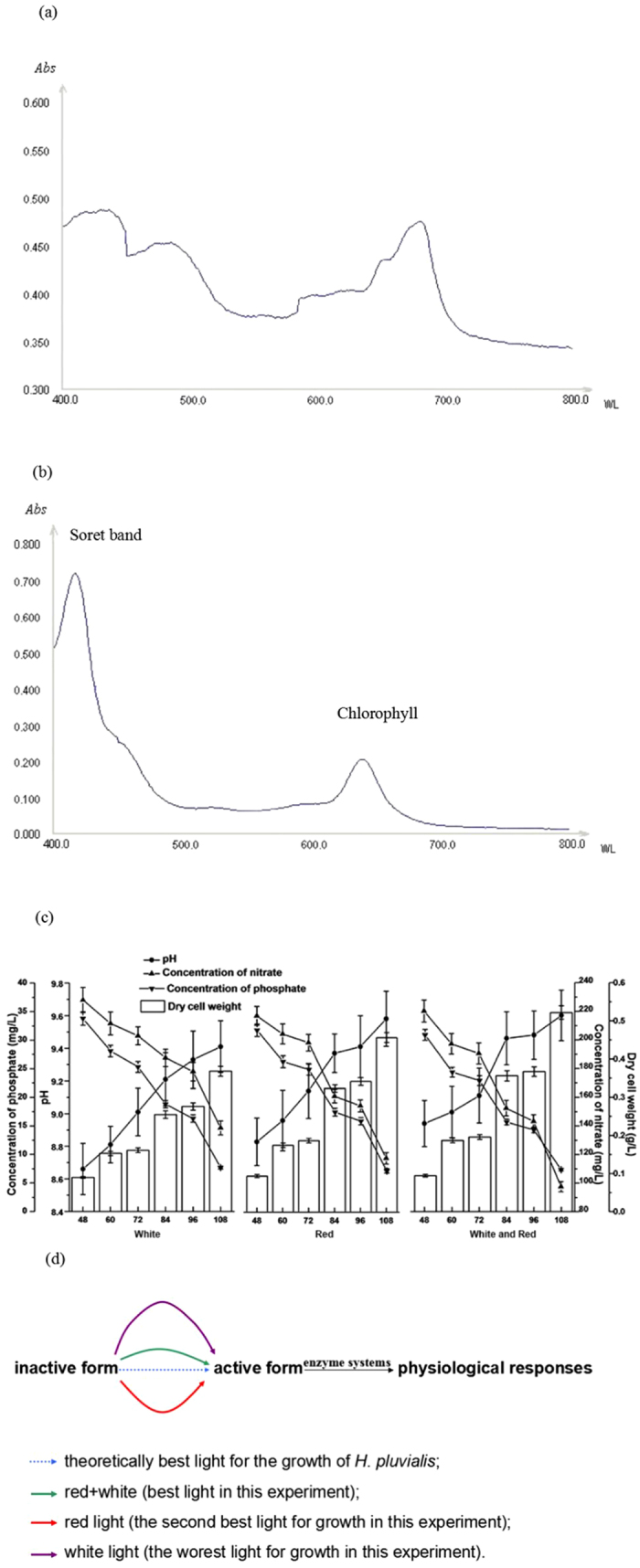
The selection of lights on green *H. pluvialis* cells. (**a**) Absorption spectra of green *H. pluvialis* cells; (**b**) Absorption spectra of chlorophyll; (**c**) Effect of lights on nutrient assimilation, pH state and *H. pluvialis* growth, values are shown in mean ± s.d; (**d**) Sketch of light effect.

The absorption wavelength of green cells of *H. pluvialis* was investigated to figure out high energy ranges (measured by wavelength) that were easily absorbed by *H. pluvialis* in the growth stage. As shown in Fig. 2a, obvious absorption peak of green cells could be obtained at 680 nm and high absorption wavelength were displayed from 400 to 500 nm and 650 to 700 nm, which were similar with chlorophyll in *H. pluvialis* (Fig. 2b). Different photosynthetic efficiency of *H. pluvialis* will present when illuminant is in the diversity. Therefore, light intensity and wavelength were investigated to reveal the enhanced effects on cell activity and cell biomass.

The role of light intensity in *H. pluvialis* refers to light saturation of cell. The rate of photosynthesis increases as the light intensity is enhanced in the initial stage. When the light intensity is high enough, the status of light saturation is presented, which lies on the appointed plant, caused by the overburdened enzymatic reaction. Cells absorb energy from light and then a series of enzymes in living cells attempt to “resolve” the energy. When the light saturation appears, it is concluded that enzymatic chemical organs in cells are in overload (Fig. 2d). Thus, light intensity at 2500 lx for this strain of *H. pluvialis* was set as the optimum ( Supplementary Fig. 1).

To evaluate the roles of emission wavelengths acted in cell growth phase, specific growth rate and dry cell weight were measured. The short-term specific growth rate could be regarded as index to represent cell activity and dry cell weight to cell biomass. Therefore, in the exponential growth phase, relationship among dry cell weight and cultivation conditions of pH, concentration of nitrate and phosphate were figured out.

As shown in Fig. 2c, different trends of cultivation conditions and dry cell weight were obtained. The dry cell weight increased rapidly by mixed-light with the value of 0.522 ± 0.018 g/L, which was increased 14.4% to red light and 41.8% to white light, respectively. Furthermore, the consumption of nitrate in this stage decreased significantly by mixed-light, suggesting that mixed-light of white and red impelled cell to absorb nutrients for building up new tissues, which could be obtained from the dry cell weight. However, the values of consumption of phosphate showed opposite results that significant change of phosphate concentration was displayed in the white light. In the previous research, we found that phosphate concentration rose suddenly when the nutrients were depleted and proposed the possibility of autopepsia^14^. The phenomenon of autopepsia displayed in every stage of cell growth and the changes of phosphate in this research made possible that mixed-light and red light promote the cell biomass in a short time, while against cell activity in a long time. This was also one of the reasons that enhanced effect of dry cell weight was less significant at green growth stage than at exponential growth phase by mixed-light. As shown in Fig. 2d, mixed-light of red and white was the best light in this experiment to induce *H. pluvialis* to transform to “active form” with the highest photosynthetic efficiency and then the physiological responses were easily occurred with the enzyme systems. Theoretically, optimal light for metabolism of green plants exists. The selection of the lights in microalgae cultivation was in the purpose to maximize the photosynthetic efficiency. In this research, mixed-light of red and white was the best light, closest to optimal light, to enhance the cell biomass.

The specific growth rate was a well index to reveal the effect of emission wavelengths on cell biomass and cell activity. Specific growth rate in Table 1 showed that mixed-light of white and red was more propitious for cell growth than single light with the value of 0.683 at exponential growth phase and 0.361 at green growth stage. The specific growth rate decreased largely with the cultivation time went. The major reason was the insufficiency of nutrients, which reached 7–8 mg/L of phosphate and 90–140 mg/L of nitrate at 108 h (Fig. 2c). The initial high nutrient concentration would inhibit the cell growth. Therefore, fed-batch culture was essential for the purpose of high biomass.

### Fed-batch culture by pH

**Figure 3:**
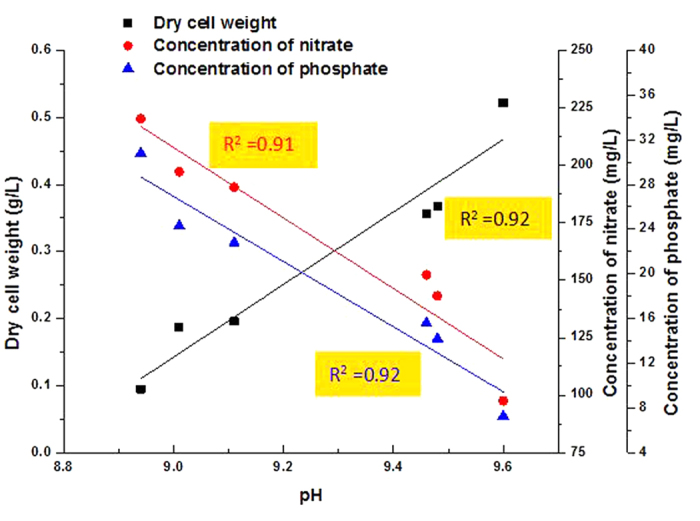
Fed-batch culture of *H. pluvialis* growth. (**a**) Linear relationship among pH, dry cell weight and nutrient concentration. Values are mean ± s.d.

In order to enhance the biomass of *H. pluvialis*, fed-batch culture was adopted. The difficulties of fed-batch culture of *H. pluvialis* are the determination of feeding time, medium composition and the way to fed-batch, which will impede *H. pluvialis* growth in an improper manner. Interestingly, Fig. 2c revealed the relationship between pH and nutrients (nitrate and phosphate), which was an available index in cultivation processes. As shown in Fig. 3a, the pH displayed linear relation with the concentration of nitrate and phosphate in the exponential growth stage, which made it possible to operate fed-batch according to the changing pH, namely the concentration of acetate in this stage and the growth yield of *H. pluvialis* at mixed-light on nitrate and phosphate was 3.50 g g^−1^ and 18.65 g g^−1^, respectively. Thus, the changes of pH might be the good signal to reflect the conditions of dry cell weight and concentration of nitrate and phosphate. As shown in Supplementary Fig. 2, the dry cell weight increased to 1.63 ± 0.07 g/L and a 20.8% increase in the specific growth rate was obtained as the addition of fed-batch media using pH as a detector, which led the conclusion that this method provided enough nutrients in the exponential growth phase of *H. pluvialis.*

### Extraction of astaxanthin from H. pluvialis during the transformed cultivation environment

In the initial accumulation stage, the modified enzymes of cell wall act to resistant the stressed environment, causing the thickness of cell wall. While the conditions of cell wall responding to the high light in the initial accumulation stage are unknown. Thus, the extraction yield of astaxanthin from the none-cell disruption of *H. pluvialis* was measured to evaluate the damage of cell wall.

**Figure 4:**
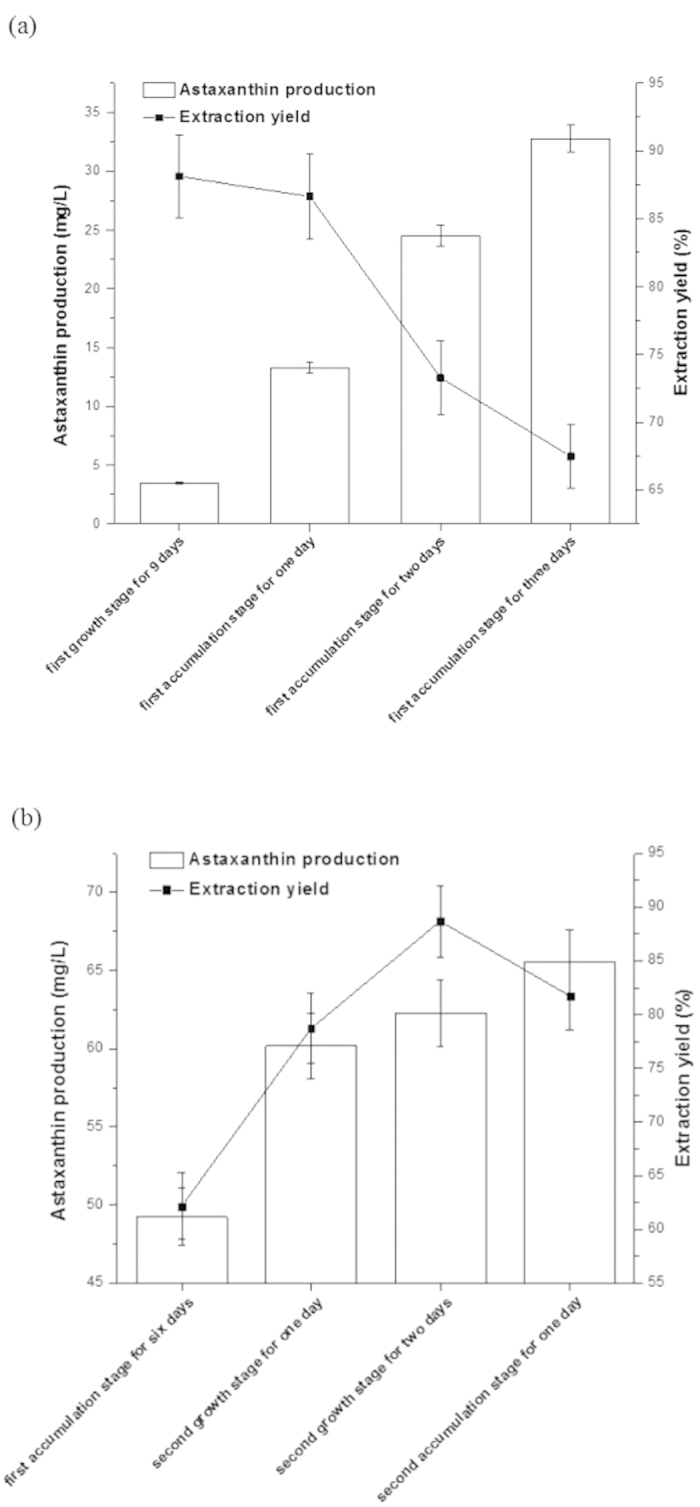
Astaxanthin production by extraction reagents. Values are mean ± s.d. (**a**) Astaxanthin production from none wall-broken cell at days in first growth and accumulation; (**b**) Astaxanthin production from none wall-broken cell at days between first and second accumulation stage.

As shown in Fig. 4a, the extraction yield of astaxanthin reached the maximum at the initial accumulation stage for one day, suggesting that the cell wall was damaged under the high light intensity and the effect of modified enzymes was not obvious. The thick and solid cell wall was formed as the stressed environment kept on, which was the major reason for the low extraction yield. The step to induce *H. pluvialis* to return to green cells was operated. Extraction yield in Fig. 4b showed that inducing the germination of *H. pluvialis* relaxed the structure of cell wall, then increased extraction yield. In the process, the degradation of astaxanthin was not significant. The second accumulation stage was opened to increase the amount of astaxanthin. And the extraction yield decreased, while the extracted astaxanthin increased. Thus, converting the stressed environment to suitable growth environment will avoid the situation of decreased extraction yield. The later turgor pressure will also push on cell wall and the relaxation of cell wall was changed.

### Determination of intracellular adenosine triphosphate(ATP) and reactive oxygen species(ROS) concentration during the transformed cultivation environment

**Figure 5:**
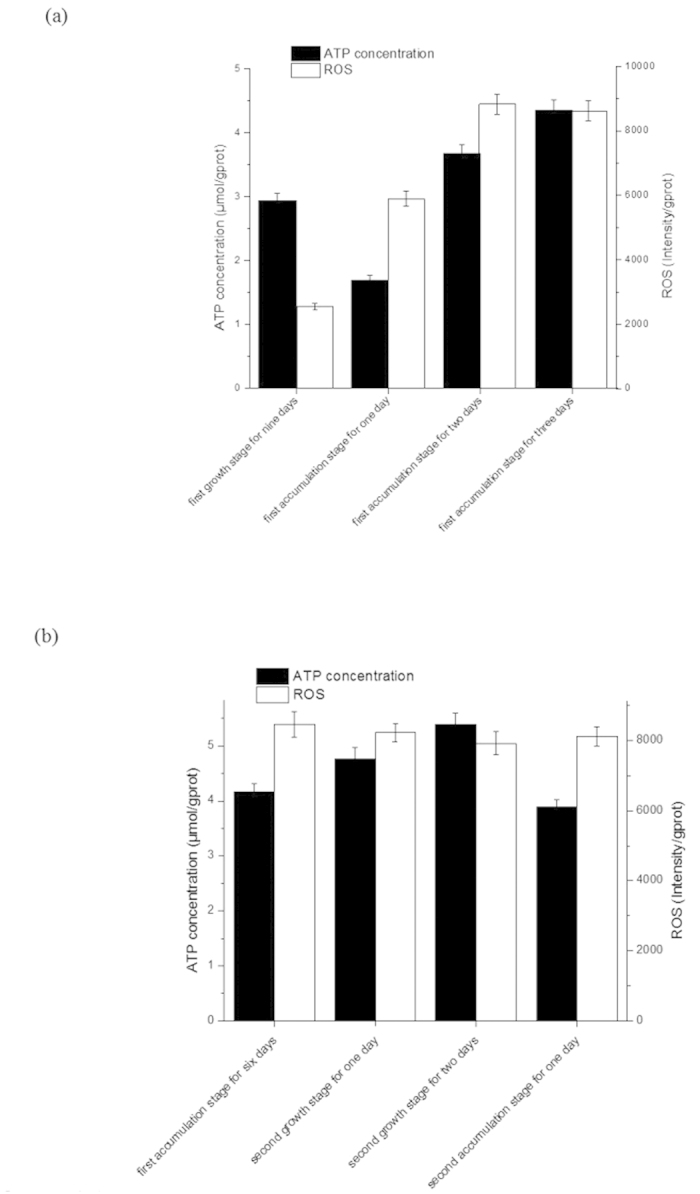
Intracellular ATP) and reactive ROS concentration during the transformed cultivation environment. Values are mean ± s.d. (**a**) Transformed cultivation environment from first growth stage to first accumulation stage; (**b**) Transformed cultivation environment from first accumulation stage to second accumulation stage.

ATP plays a significant role in the growth of microalgae and the production of the target metabolites. Nine days was set as the first growth stage and 6 days was set as the first accumulation stage according to our previous research^6^. As shown in Fig. 5a, ATP concentration decreased in the first day and then increased significantly at the expense of protein degradation. A high ATP demand was badly needed to synthesize astaxanthin when *H. pluvialis* grown on high environmental stress. In this period, the level of ROS increased as signaling molecule that potentially induce defense pathways, namely the process of astaxanthin accumulation. Then ROS concentration kept at an unaltered high level at the last stage (Fig. 5b), while ATP concentration rose significantly at the second growth stage with the supplement of nutrients.

### Further optimal extraction of astaxanthin from H. pluvialis

For the further study to evaluate the effect of repeated cultivation on non-cell disruption, the optimal cell wall-broken method was supposed to compare with non-cell disruption of *H. pluvialis* by the proposed method.

As shown in Supplementary Fig. 3, the wall-broken rate was 40.3% at the frequency of 50 Hz and rotation rate of 23,000 rpm for 3 min with the astaxanthin concentration at 33.3 mg/L. The maximum value of wall-broken rate was obtained after 6 min and the value was not increased after that. The wall-broken time was set at 6 min by high speed homogenization method.

**Figure 6:**
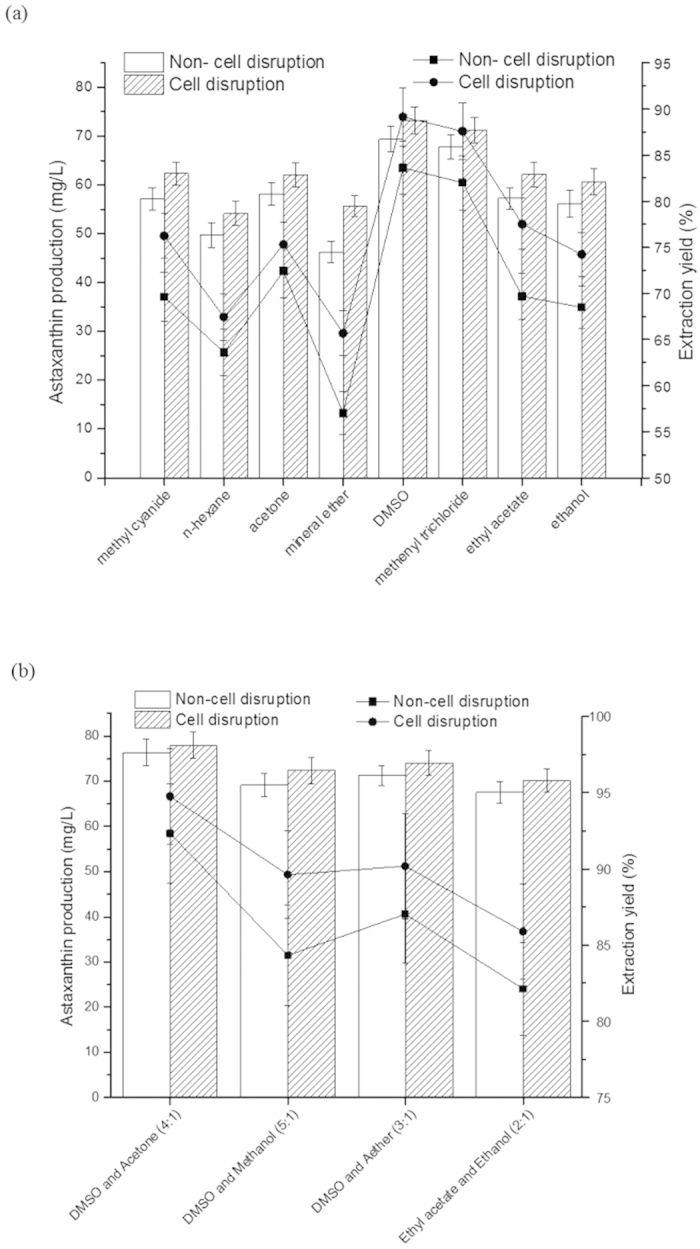
Astaxanthin production by extraction reagents. Values are mean ± s.d. (**a**) Astaxanthin production to two different treated *H. pluvialis* with single reagent; (**b**) Astaxanthin production to two different treated *H. pluvialis* with mixed reagents. Line represents extraction yield and cylindricality is astaxanthin production.

Astaxanthin is soluble in organic solvent. Thus, several organic reagents were used to extract astaxanthin from *H. pluvialis* without cell disruption, compared with the broken algae. As shown in Fig. 6a, DMSO was the optimum single extraction reagent with the astaxanthin production of 73.21 ± 2.75 mg/L to broken algae and of 70.37 ± 2.64 mg/L to no broken algae. Seldom difference was found in the final yield of astaxanthin with the different treated algae. DMSO and ethyl acetate lack hydrophilicity, resulting in the poor ability to enter into the cells. Combined with high polar reagent such as acetone, ethanol, aether and methanol, DMSO and ethyl acetate were easier to get in touch with the astaxanthin, especially in the algae without cell disruption. The effect of different ratios of mixed reagents on astaxanthin production was studied ( Supplementary Fig. 4). As shown in Fig. 6b, no significant difference could be obtained by the mixed reagents of DMSO and acetone with the astaxanthin production of 76.38 ± 2.95 mg/L and extraction yield of 92.32 ± 3.24 compared to non-cell disruption by repeated cultivation.

## Discussion

Production of metabolites from microalgae has emerged as the promising alternative in the commercial process as human realize the value of these metabolites and the economical efficiency of algae production. The operation of cell disruption is an essential step in every production process, causing sizable cost in each company. However, it is only within the past few decades that interests in microalgae cell wall have been intensified^22^,^23^,^24^,^25^. In the growth stage, cell wall can be mechanically manipulated by expansin^26^ or pectin methyl-esterase^27^ to induce loosening and then a turgor pressure is expected if these proteins still exist in the accumulation stage. Summarized from the reported researches of cell wall for bioenergy, stress relaxation in plant cell wall and photosynthesis of microalgae, repeated cultivation is proposed in the production process. With the easy operation to transfer the cultivation conditions, the harvest of non-cell disruption is achieved, renovating the traditionally commercial microalgae production process.

In the growth stage, the new tissues and large amount of ATP are produced, resulting in pressure on cell wall to enlarge itself. While in the metabolite-accumulated stage, the new tissues were no longer synthesized and the large amount of ATP flows to synthesis of astaxanthin due to the depressed photosynthesis and the stressed nutrient concentration^28^. The amount of ATP produced by microalgae has direct effect on both biomass and cell size. The cell biomass obtained in the cultivation process can vary greatly with the composition of growth medium, environment and proportional to the amount of ATP. The major energy costs in microalgae are much the same^29^. Thus, the supplement of ATP is significant for cell to enlarge itself and theoretically the turgor pressure has positive correlation to ATP. In the microalgae growth stage, turgor pressure is decreased by the stress relaxation of cell wall until the yield threshold for cell expansion is reached^25^. The cell wall will remain unchanged in the succeeding accumulation stage with the less pressure until microalgae growth occurs again. Thus, the increase of ATP concentration at second growth stage will be beneficial to the “wall loosening” (Fig. 5b).

**Figure 7:**
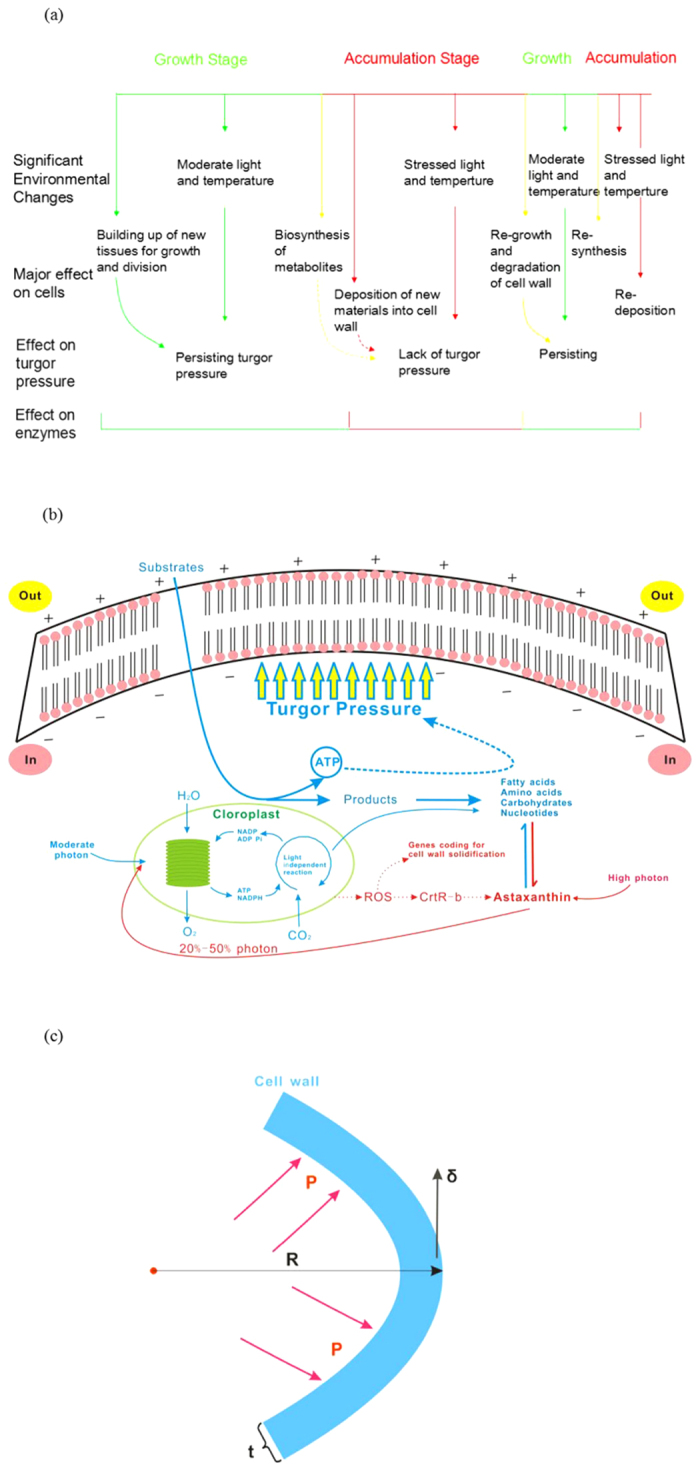
Effect of repeated cultivation on cell wall. (**a**) *H. pluvialis* is responsible for growth in the moderate environment, accompanied with large amount of ATP to engender turgor pressure. Astaxanthin accumulation occurs when the environment converts to stress to absorb the redundant photon. Enzyme in growth stage is present in the initial accumulation stage. The cell wall gets solid in the succedent accumulation stage once the accumulated enzymes start to work. Blue line represents growth process, red line is accumulation process and yellow line is period between growth and accumulation process; (**b**) The relationship among stressed conditions, cell growth and division and metabolite synthesis. Blue line represents growth process and red line is accumulation process; (**c**) Turgor pressure acts on cell wall.

The reported results have revealed that two enzyme systems exist in *H. pluvialis* life cycle, one in green growth period and the initial accumulation stage, the other in the later stable astaxanthin accumulation stage^28^. Thus, a delay of enzymatic work is presented in the conversion of the cell morphology (Fig. 7a). The immune response launched to modulate plant cell resistance under stressed light, followed by changes occurring in ROS metabolism (Fig. 5b), such as deposition of new materials into cell wall, biosynthesis of metabolites and activation of hormonal signalling^30^. New idea has been advanced that ROS is harnessed by growing cells to loosen their cell wall^31^,^32^. The high level of ROS at second growth stage will also be beneficial to the “wall loosening” (Fig. 5b). The selection of light was used as the “switch” in the process of this transformation to produce corresponding ATP and ROS for metabolism^33^.

Therefore, the repeated cultivation is proposed in this process based on the structure and mechanics of cell wall and photosynthesis (Fig. 7b). The structure of cell wall undergoes significant changes when respond to different cultivation environment. Hypotheses of turgor driven creep and new material deposition are widely accepted in the explanation of cell wall growth. The cross-links in cell wall will be broken or rebuild in the growth stage by Spencer’s deviatoric stress and the new material will insert into cell wall for the expansion manipulated by turgor pressure^34^. Typical plant cell turgor pressures result in 10–100 MPa of tensile stress in the wall, which is applied to regulate cell wall for easy extraction^35^. The force situation of cell wall is presented in Fig. 7c. The tensile stress in the cell wall is:


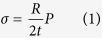


where *R* is the radius, *P* is the internal pressure and *t* is the cell wall thickness. The strain ε is:


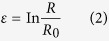


where *R*_*0*_ is the initial radius and according to general stress-strain equation. The elastic property of the cell wall is:


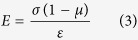


Assuming Poisson’s ratio *μ* = 0.5^35^. The relationship among *E, P*, and *t* will be displayed as follow:


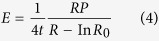


thus, the elastic property of cell wall has positive correlation with internal pressure and negative with cell wall thickness. When cells respond to stressed environment, the cells solidify the material of cell wall and form thick cell walls, then the re-emerging turgor pressure will decrease the relaxation of cell wall. Thus, combined with equation (4), E will increases when turgor pressure occurs in the accumulation stage that the work of enzymes turns to green stage. In the stressed environment, the amount of intermediates of carbohydrates and fatty acids increase, which are the materials for astaxanthin accumulation. The induced astaxanthin then protect cell by preventing or decreasing the redundant light and the cell growth and division are inhibited due to the decrease of amino acids for new tissues and the thickness of cell wall (Fig. 7b).

Numerous studies have concluded to investigate the cultivation of *H. pluvialis* on new techniques and methods such as one-step production, attached cultivation, immobilized biofilm cultivation and kinetic models for astaxanthin production, while the industrial application was not desirable^36^,^37^,^38^,^39^. Created bioreactors, new operating system and the application of computer technique have a certain requirement for equipment and operators that restricts their application timely and widely. Repeated cultivation is a common approach with a wide application. Cell disruption is removed in industrial process of microalgae with the same extraction yield as two-stage process and the production of astaxanthin are higher than one-stage process. Only the cultivation cycle is added as the “natural energizer” in *H. pluvialis.* Unlike the traditional disruption methods, repeated cultivation does not need the high specific energy consumption like mechanical methods, long treatment time and large demand for chemicals like non-mechanical methods^3^,^5^,^9^,^10^. Furthermore, the production yield is same as them. It was meaningful for repeated cultivation that it could be widely used in industry for its easy operation, low requirements for equipment and environment, and satisfactory astaxanthin production.

Research of repeated cultivation has broad implications in microalgae cultivation, not only in extraction of production, but also in control of cell growth and improvement of bioenergy. *H. pluvialis* showed growth property that similar to stored growth. The cell becomes active when pressure is reestablished^40^. The turgor pressure plays a significant role in cell growth and division and thus it need to be sensed and transduced^41^. The regulation of turgor pressure in this research provides a trend for a single cell to grow or divide by changing the cell wall structure. The degradation of the carbohydrate polymers of algae cell walls will provide necessary substrates for fermentation to ethanol and thus the researches of manipulation of cell wall genes presented flourishing in past few years^1^,^23^. The repeated cultivation changes the cell wall which makes possible that the degradation of the treated cell wall easily occurs^36^.

This study proposed repeated cultivation in *H. pluvialis* growth to achieve the goal of non-cell disruption and the result proved the feasibility. Manipulation the cell growth by regulating the conditions between growth and stressed environment to product the repeated pressure and exploit the delayed enzyme effect, working on the cell wall, was the key in this process. This repeated cultivation process cuts the cost of algal byproducts that possess huge marketable potential and thus promotes the development of algal bioenergy market in the long term.

## Materials and Methods

### Medium composition and growth conditions

*H. pluvialis* 712 was preserved in School of Food Science and Engineering, Ocean university of China. The seed medium for this strain was the revised MCM medium (pH 8.0), which consisted of (in g/L unless otherwise stated): KNO_3_ 0.6, KH_2_PO_4_ 0.02, MgSO_4_·7H_2_O 0.2, CaCl_2_ 0.04, Fe-EDTA 100 (in μL/L), microelement 100 (in μL/L). The Fe-EDTA solution (in g/L) consisted of EDTA·2Na 3.72, FeSO_4_·7H_2_O 4.17. The microelement (in g/L) consisted of H_3_PO_4_ 12.37, MnSO_4_·H_2_O 84.51, ZnSO_4_ 71.89, CuSO_4_·5H_2_O 62.42, Na_2_MoO_4_·2H_2_O 7.26, CoCl_2_·2H_2_O 4.76. While the growth medium (in g/L unless otherwise stated) for *H. pluvialis* 712 consisted of: KNO_3_ 0.3, KH_2_PO_4_ 0.04, MgSO_4_·7H_2_O 0.2, CaCl_2_ 0.08, 100 (in μL/L), microelement 100 (in μL/L), with the supply of 2.4 g/L sodium acetate. The medium was adjusted to pH 7.5 and autoclaved at 121 °C for 20 min. About 10 mL of a 15-day culture was inoculated into 100 mL sterilized growth medium in a 300-mL Erlenmeyer flask. The flask was incubated for 9 days at 22 °C at day and 16 °C at night in an incubator (QHX-250BS-III, Shanghai CIMO Medical Instrument Co., LTD, Shanghai, China). Light was provided by red and white lights at 2500 lx with a dark/light cycle of 12 h: 12 h.

### Fed-batch culture

Fed-batch culture of *H. pluvialis* was carried out by continuous feedings of nutrients, comparing with no addition of nutrients in a stable pH. The seed culture in the active state was inoculated into 300-mL Erlenmeyer flasks for cell growth. The initial pH was set as 7.5. As the cultivation time went, pH increased significant. The fed-batch medium was used as nutrient and acid supplements and the pH was prepared around 5.0. The fed-batch medium was added in the Erlenmeyer flasks to keep the pH of growth medium between 7.5 to 8.0 with use of precise pH test paper in the clean bench. This operation kept three times a day. The fed-batch medium (in g/L unless otherwise stated) consisted of CH_3_COONa 24.0, KNO_3_ 7.88, KH_2_PO_4_ 1.49 and autoclaved at 121 °C for 20 min. The rest of conditions were same as subculture and seed culture.

### Repeated cultivation

As the traditional two-stage process, the encystment of *H. pluvialis* is induced after the growth stage for 9 days. The temperature was set at 30 °C for the whole day. The light was changed to 7000 lx with the mixed-light of blue and white (3:1). The first accumulation stage lasted for 6 days. Then the cultivation environment returned to growth conditions with the red and white lights at 2500 lx and temperature at 22 °C in a dark/light cycle of 12 h: 12 h, which meant the second growth stage started. This stage lasted for 2 days with the supplement of nutrients of CH_3_COONa, KNO_3_ and KH_2_PO_4_ (ratio of 16.1: 5.3: 1) to induce the germination of *H. pluvialis.* The conditions were then transformed to the second astaxanthin accumulation stage for 1 day and the environment conditions were same as the first accumulation stage. The last growth and accumulation stages continued until the satisfactorily extracted yield achieved.

### Determination of dry cell weight, specific growth rate, protein, ATP and ROS

Cell growth was monitored by measuring the different absorbance at 680 nm and 750 nm with a UV/VIS spectrophotometer (UV-2802PC UNIC, Shanghai, China), and the dry cell weight was calculated through equation (5)^30^.


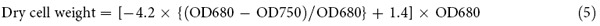


The specific growth rate of *H. pluvialis* was calculated through equation (6).


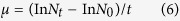


where *N*_*t*_ is the dry cell weight at the cultivation of t days (g/L), *N*_*0*_ is the initial dry cell weight (g/L).

The protein content in *H. pluvialis* was determined by the dye-binding method as described by Bradford^42^. ATP concentration was determined lumiically with the spectrometer as described by Mahro^43^ and the result was presented as μmol per g protein. ROS was measured by reagent of DCFH-DA with fluorescence spectrophotometer (F-4600, Hitachi High-Technologies Corporation, Tokyo, Japan). The excitation wavelength was set at 500 nm and the emission wavelength was set at 525 nm^44^. The amount of ROS was presented as fluorescence intensity per g protein.

### Analysis of the absorption of green cells and chlorophyll

The measurements of absorption spectra of *H. pluvialis* cells and chlorophyll were performed by a UV/VIS spectrophotometer (UV-2802PC UNIC, Shanghai, China). The absorption wavelength of *H. pluvialis* cells was scanned from 400.0 nm to 800.0 nm. Chlorophyll was extracted by 5% KOH and 30% (v/v) methanol at 65 °C and the scanned wavelength was from 400.0 nm to 800.0 nm^45^.

### Analysis of astaxanthin concentration

The astaxanthin concentration was determined photometrically^45^. The red cyst cells were collected by centrifugation at 4800 r/min for 15 min. To remove the chlorophyll in *H. pluvialis*, the pellet was redissolved in a solution of 5% KOH and 30% (v/v) methanol at 65 °C three times until the supernate’s color was empty. Then it was washed three times to scour off the residual alkali. Cell wall disruption is performed by high speed dispersion homogenizer (JY92-II Ningbo Scientz Biotechnology Co., LTD, Ningbo, China) at 50 Hz and 23000 r/min. Miscible liquids were then added to the broken cell or non-broken cell. The operation was repeated until the pellet was almost colorless. The red supernatant was collected to measure the absorbance at 490 nm. The astaxanthin concentration was calculated through equation (7).


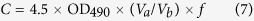


where *C* is the astaxanthin concentration (mg/L), *V*_*a*_ is the volume of extracts (mL), the *V*_*b*_ is the volume of culture sample (mL), *f* is the dilution ratio of measuring the absorbance.

Measurements were taken in triplicate for each sample in the research except the measurements of absorption spectra of *H. pluvialis* cells and chlorophyll.

## Additional Information

**How to cite this article**: Sun, H. *et al.* Repeated cultivation: non-cell disruption extraction of astaxanthin for *Haematococcus pluvialis*. *Sci. Rep.* **6**, 20578; doi: 10.1038/srep20578 (2016).

## Supplementary Material

Supplementary Information

## Figures and Tables

**Figure 1:**
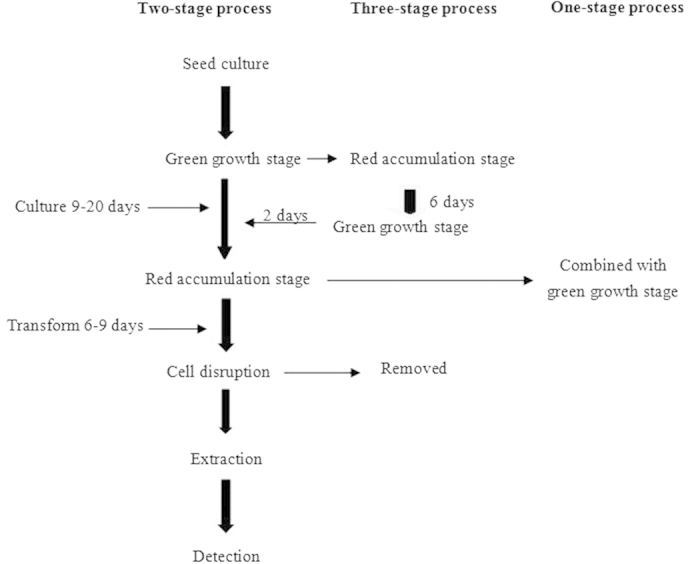


**Table 1:** Conditions of indexes of *H. pluvialis* in exponential and green growth phase.

Indexes	Conditions of*H. pluvialis*					
	Exponential growth phase			Green growth phase		
	White	Red	Mixed	White	Red	Mixed
Specific growth rate (d^−1^)	0.565	0.635	0.683	0.345	0.351	0.361
Changes of dry cell weight (g/L)	0.279	0.363	0.427	0.701	0.742	0.816
Consumption of nitrate (mg/L)	89.5	99.2	122.8	165.3	187.9	204.8
Consumption of phosphate (mg/L)	26.1	24.6	23.5	*—*	*—*	*—*
