# Supplementary material for: Repeated cultivation: non-cell disruption extraction of astaxanthin for Haematococcus pluvialis
Source: Sci Rep. 2016 Feb 3;6:20578. doi: 10.1038/srep20578 (PMC4738327; doi:10.1038/srep20578)
Supplement: Supplementary Information [file srep20578-s1.doc]

## Supplementary Information

**Repeated cultivation: non-cell disruption extraction of astaxanthin for *Haematococcus pluvialis***

**Han Sun, Bin Guan*, Qing Kong*, Zhaoyan Geng, Ni Wang**

School of Food Science and Engineering, Ocean University of China, Qingdao, Shandong 266003, China

Supplementary Figure S1: Effect of light density intensity on *H. pluvialis* growth. Line represents specific growth rate and cylindricality is dry cell weight. Values are mean±s.d.

Supplementary Figure S2: Effect of fed-batch culture according to the linear relationship on *H. pluvialis* growth. Values are mean±s.d.

Supplementary Figure S3: Wall-broken rate of spore by breaking the wall with organization homogenate machine. Values are mean±s.d.

Supplementary Figure S4: Effect of different ratios of solvents on extraction of astaxanthin from *H. pluvialis.* Values are mean±s.d.
